# Metal-induced aggregation of valine capped gold nanoparticles: An efficient and rapid approach for colorimetric detection of Pb^2+^ ions

**DOI:** 10.1038/s41598-017-08847-5

**Published:** 2017-08-24

**Authors:** E. Priyadarshini, N. Pradhan

**Affiliations:** 1 Academy of Scientific and Innovative Research, CSIR-Institute of Minerals and Materials Technology, Bhubaneswar, 751013 India; 2 Environmental and Sustainability Department, CSIR-Institute of Minerals and Materials Technology, Bhubaneswar, 751013 India

## Abstract

In this study, we report a novel application of valine-capped gold nanoparticles for colorimetric and visual detection of lead ions. The –COO^−^ group of the hydrophobic valine molecules present efficient electrostatic repulsion resulting in generation of stable, well-dispersed and size-controlled GNPs. The GNPs were highly selective for Pb^2+^ ions and showed visible colour change in the assay mixture on addition of solution containing lead ions. Interestingly, a decrease in the intensity of original SPR peak at 530 nm was observed, with concomitant appearance of a new peak at longer wavelength due to agglomerated GNPs. The free –COO^−^ groups on GNP surface interacted with Pb^2+^ and ion-dependent chelation mechanism lead to cross-linking of particles and subsequent agglomeration. Binding of Pb^2+^ ions and valine-capped GNPs occur in a stochiometric ratio of 1:2. The GNPs displayed colorimetric sensing in the range of 0 to 100 ppm concentration with a very high selectivity towards lead even in the presence of other metal ions. The minimum detection limit (MDL) for Pb^2+^ was 30.5 µM. We anticipate that these valine-capped GNPs may be employed for lead detection in polluted water/wastewater through a cost-efficient, one-step assay protocol as it does not require additional functionalization with specific ligand molecules.

## Introduction

Lead (Pb) is considered as a major and dangerous ubiquitous heavy metal pollutant and is associated with serious health problems when present in the aquatic system and human body^1^. In the last decade, a number of anthropogenic and industrial activities such as smelting, use of pesticides, battery production, landfill leaching have significantly contributed towards Pb contamination in the environment^2^. Pb^2+^ is a bio-accumulative and potent neurotoxin that cause potential damage to normal cellular functions, affects signal transduction, gene expression and calcium-mediated cellular processes^3–5^. The maximum acceptable limit of Pb^2+^ is 0.05–0.10 mg/L in waste water; however, Pb^2+^ concentration has been reported to reach to 250 mg/L in industrial waste water^6^ and is becoming a major environmental concern. Owing to this, there is a huge demand for developing a rapid assay method that would allow detection of Pb^2+^ ions.

A number of analytical techniques like atomic absorption spectroscopy, mass spectroscopy, fluorescence, electrochemical and DNAzyme based methods are being currently used for determining Pb^2+^ ion^7–11^. However, the development of a simple and rapid colorimetric method for Pb^2+^ detection could circumvent the use of expensive and tedious analytical instruments. Gold nanoparticles (GNPs) have been widely investigated for their potential ability to detect Pb^2+^ ions. They allow colorimetric detection of metal ions because of their intriguing optical properties. Depending on the size, shape and aggregation state, GNPs show distinct colour and concomitant shifting of the absorption spectra^12^. The detection of metal ions using gold nanoparticles is fundamentally based on the aggregation of GNPs induced by binding of metal ions to GNP surface, resulting in corresponding chromatic change in colour and shifting of plasmon peaks. Based on this principle, GNPs functionalized calixarene^13^, pentapeptide cysteine-alanine-leucine-asparagine-asparagine(CALNN)^14^ and DNAzyme based methods^15, 16^ have been used for detection of Pb^2+^ ions. Although these aforesaid assay methods have high sensitivity and selectivity for Pb^2+^ detection, they are tedious and time consuming. These require additional steps of modification of the GNP surface with Pb^2+^ specific functionalizing ligand after the synthesis.

In the present work, we developed a rapid, sensitive and label-free colorimetric method for detection of Pb^2+^ ions using as-synthesized GNPs. The strategy is based on selective metal induced agglomeration of the monodispersed GNPs leading to a change in colour of colloidal GNP solution and changes in absorption spectra. On adding Pb^2+^ ions to the GNP solution, Pb^2+^ binds to the free –COO^−^ groups of valine capped GNPs with high affinity resulting in destabilization of negative charges on GNP surface. Electrostatic destabilization leads to agglomeration of individual particles resulting in change in colour of colloidal solution as well as surface plasmon resonance (SPR) peak. Detection of Pb^2+^ ions could be comprehended within few minutes, with a minimum detection limit (MDL) of 20 ppm. The approach involves visual detection without the use of electrolytes or transducers. Hence, the method allows rapid and cost effective colorimetric detection of Pb^2+^ ions. Furthermore, in comparison to other colorimetric approaches reported, the present method avoids the use of additional functionalizing agent. The method, thus presents the advantage of using as-synthesized GNPs, without the requirement of two step tedious procedure of modification/ functionalization of GNP surface. The optical sensor developed herein presents a cost-efficient yet rapid tool for Pb^2+^ monitoring and holds potential applicability as next generation colorimetric tools for Pb^2+^ detection.

## Experimental Section

### Materials

Hydrogen tetrachloroaurate (HAuCl_4_.3H_2_O) was purchased from Sigma-Aldrich Co. (USA). All other chemicals used in the study were purchased from SRL, India. All the chemicals were of analytical grade and used as received. Standard stock solutions of metals were prepared in ultrapure Milli-Q water and stored at room temperature for further use.

### Preparation of gold nanoparticles

The working concentration of valine in the study was 0.1 M, which was prepared by appropriately diluting the stock solution in MilliQ water and adjusting the pH of the solution with dilute NaOH. 100 ml of 0.1 M valine solution was boiled with continuous stirring. HAuCl_4_.3H_2_O (stock solution of 100 mM) was added to the boiling valine solution such that the final concentration of gold salt was 1 mM. The solution was boiled with continuous stirring till a change in colour of the solution from slight yellow to intense violet was observed. The solution was then cooled and aliquots of the solution were sealed and stored at room temperature and refrigerated conditions, to investigate the effect of aging on stability of synthesized GNPs.

To analyze the effect of pH of valine solution, the GNP synthesis was conducted with valine solution of different initial pH (in range from 6 to 10), prior to boiling. The pH that favoured stable synthesis of GNPs was chosen as the optimized pH for subsequent experiments. All other experimental analysis were conducted using colloidal GNP solution prepared using this method, without any further modifications. Effect of stability of synthesized valine-capped GNP was analyzed by adding NaOH at different concentration to the synthesized GNP solution and recording the corresponding change in absorption spectra.

### Characterization of gold nanoparticles

UV-Vis spectra in the range of 200–800 nm were recorded using CECIL UV-Vis spectrophotometer. Dynamic Light Scattering (DLS) analysis was performed using a Dawn Heleos II (Wyatt) system operating at 658 nm wavelength, at a scattering angle of 90°. The values of hydrodynamic radius (R_h_) reported in the paper are the mean of four independent measurements. Transmission electron microscopy (TEM) analysis was done using a FEI, TECNAI-G2 TEM microscope, 20-TWIN operating at 200 kV equipped with a GATAN CCD camera. Sample for TEM analysis was prepared by coating a drop of GNP solution on a carbon coated copper grid followed by drying.

### Detection of metal ions by synthesized gold nanoparticles

The valine capped GNPs were investigated for their ability to detect heavy metal ions that are generally present in waste water as pollutant. The heavy metal ions used in the study were Cu^2+^, Ba^2+^, Hg^2+^, Cr^3+^, Pb^2+^, Zn^2+^, Cd^2+^, Sb^3+^, As^3+^ and Ni^2+^. For metal detection study, synthesized GNPs were treated with 100 ppm of respective metal solutions in the ratio of 1:2 respectively and changes in the optical property was recorded at regular intervals.

The assay solution showed significant changes when treated with Pb^2+^ ion and hence we further analyzed the sensitivity of the GNPs over a range of Pb^2+^ ion concentration. UV-Vis Absorbance spectroscopy and DLS analysis of the treated solution were performed. Metal interference was studied by simultaneously treating GNPs with 100 ppm of Pb^2+^ ions and 100 ppm of different metal ions (Cu^2+^, Ba^2+^, Hg^2+^, Cr^3+^, Zn^2+^, Cd^2+^, Sb^3+^, As^3+^, Ni^2+^) separately. All detection experiments were conducted in three separate batches with triplicates in each batch to ensure reproducibility.

## Result and Discussion

### Synthesis and stability of gold nanoparticles

**Figure 1:**
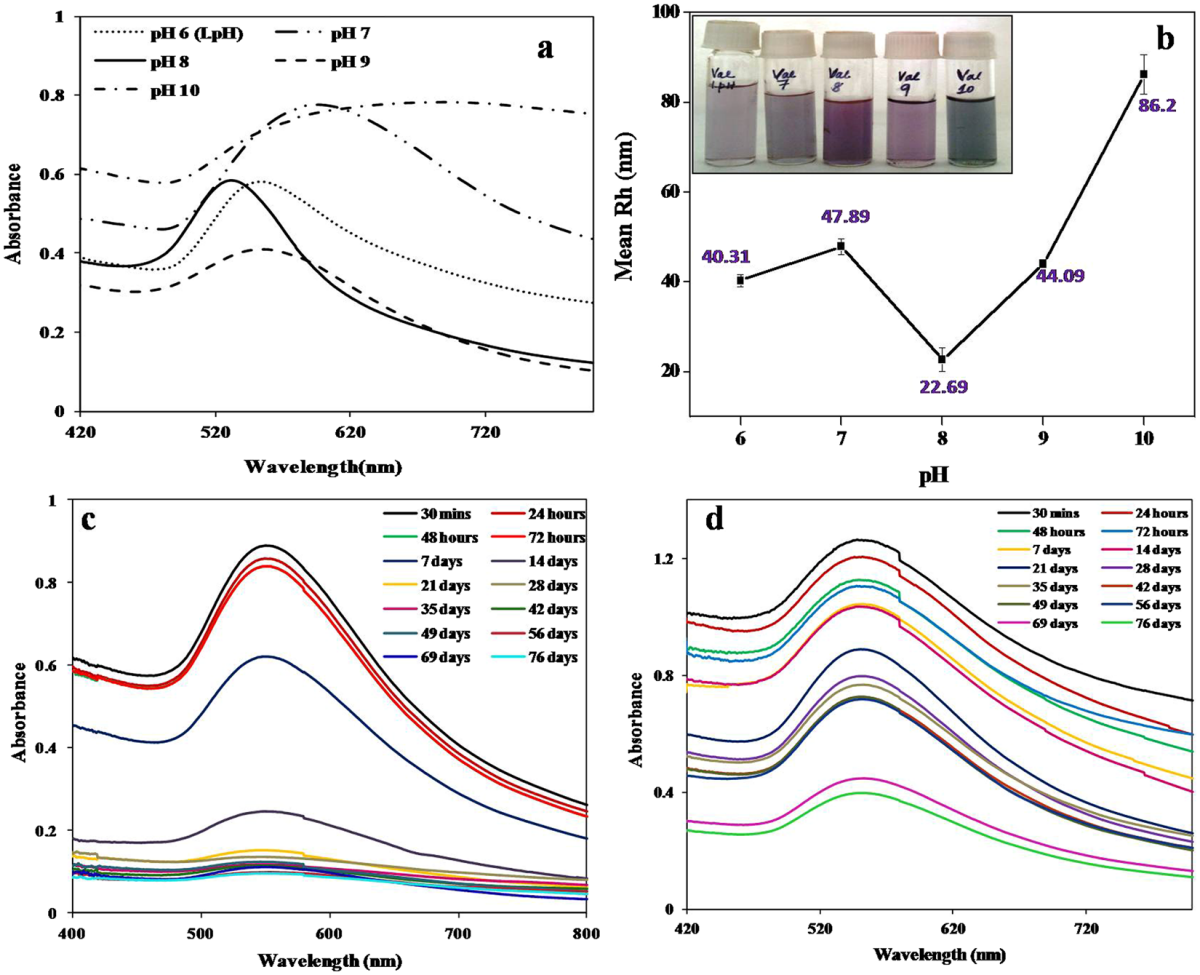
(**a**) UV-Vis Spectra depicting the effect of pH on Valine- GNP synthesis. (**b**) Effect of pH on the hydrodrodynamic radius (R_h_) of the synthesized valine-GNPs. (**c**) UV-Vis Spectra depicting stability of Valine- GNPs at room temp and (**d**) under refrigeration.

We used valine, a hydrophobic amino acid, for the synthesis of GNPs. Valine acted as both reducing and stabilizing agent in the synthesis of highly stable GNPs. Colloidal GNPs were synthesized using valine solution of different pH, ranging from 6 to 10. Figure 1a shows the absorption spectra of valine capped GNPs synthesized at different pH after an hour of formation. UV-visible spectra showed significant difference in pattern. The SPR peak at pH 8 was narrowest compared to other pH. Furthermore, broadening and red shift of peaks were observed at higher pH, which is evidence of agglomeration of GNPs at highly basic pH^17^. Agglomeration of particles above pH 9 may be because of the weak interaction between –NH of valine and Au° at alkaline pH^18^. The R_h_ values obtained from DLS analysis was in concurrence with the UV-Vis spectral data (Fig. 1b). The GNPs synthesized at pH 8 were smallest in size with average R_h_ of 22.69 ± 2.64 nm. Those synthesized at other pH were larger in size, as indicated by the red shift of SPR peak in UV Vis spectra.

Stability is an important concern in nanoparticle based applications. We therefore analyzed the stability of GNPs synthesized at pH 6 to 10. It was observed that except for particles synthesized at pH 8, GNP synthesized at all other pH precipitated within 24 hours of synthesis. This demonstrated high stability of valine-capped GNPs synthesized at pH 8.

Further study on the stability of valine-capped GNPs showed that GNPs were more stable under refrigerated condition compared to at room temperature (RT) (Fig. 1c and d). The refrigerated valine-capped GNP solution was stable for several weeks and retained the initial colour of colloidal GNP, whereas the aliquot stored at RT showed precipitation after 2 weeks of storage.

### Kinetics of valine-capped gold nanoparticle synthesis

The valine-capped GNPs synthesized at pH 8 were most stable and of smallest size. Hence all further experiments were conducted at that particular pH (pH 8).

**Figure 2:**
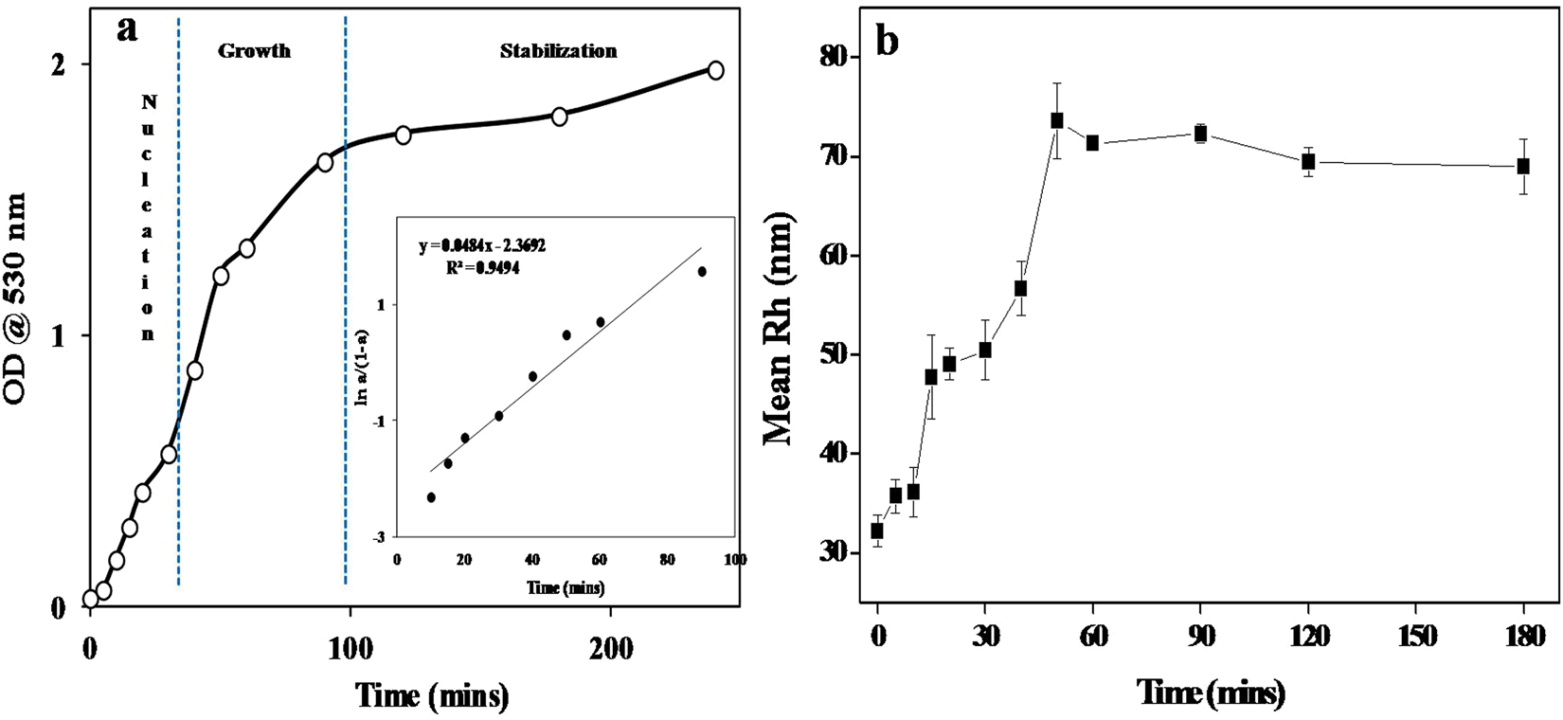
(**a**) Kinetics of Valine-GNP synthesis (pH 8) (**b**) DLS Analysis.

Based on the experimental results, we have put forth an analytical kinetic model for GNP synthesis using valine. Analysis suggested that the synthesis process follows an autocatalytic reaction model. Autocatalysis is characterized by the sigmoid nature of the kinetics graph, obtained by plotting the absorption intensity of SPR peak at 530 nm as a function of progressing reaction time (Fig. 2a). Sigmoid plot implies that a slow nucleation phase is followed by a rapid growth phase. During the initial nucleation stage, seed particles are formed which with progressing reaction time favour rapid reduction and particle growth^19^. Measurement of R_h_ of synthesized particles at consecutive time intervals also confirmed the autocatalytic mode of reaction (Fig. 2b). This was evident by a gradual increase in particle size with increasing time. The R_h_ of particles was seen to increase from 35.73 ± 1.69 nm to 73.55 ± 2 nm in the first 50 minutes of reaction and remained stable thereafter, indicating efficient capping by the valine molecules.

A quantitative measurement of the reaction rate of valine-capped GNPs synthesis was determined by plotting *ln*[*a*/(*1* − *a*)] versus time (where *a* = [*OD*(*t*)/*OD*(*∞*)]; *OD*(*t*) and *OD*(*∞*) are the optical density/absorbance at time t and ∞ respectively)^20, 21^. The plot gives a straight line curve, the slope of which is the rate constant (k_obs_) of the reaction. In the present case k_obs_ value of 0.048/min was obtained (Inset in Fig. 2a).

### Mechanism behind valine-capped gold nanoparticle synthesis

On addition of HAuCl_4_ to valine solution, redox reaction occurs reducing Au ions to Au, which subsequently acts as seed particles and assists in further nucleation and growth of particles. Valine acts as the sole reducing and stabilizing agent. The amine groups of valine molecule coordinates with Au ions via weak covalent linkage as reported^22^. The formation of –NH-Au^0^ bond renders electrostatic stabilization to the synthesized particles. Additionally, the presence of free negatively charged carbonyl (–COO^−^) on GNP surface, provides electrostatic repulsion, stabilizing the particles and preventing particle aggregation in aqueous system.

**Figure 3:**
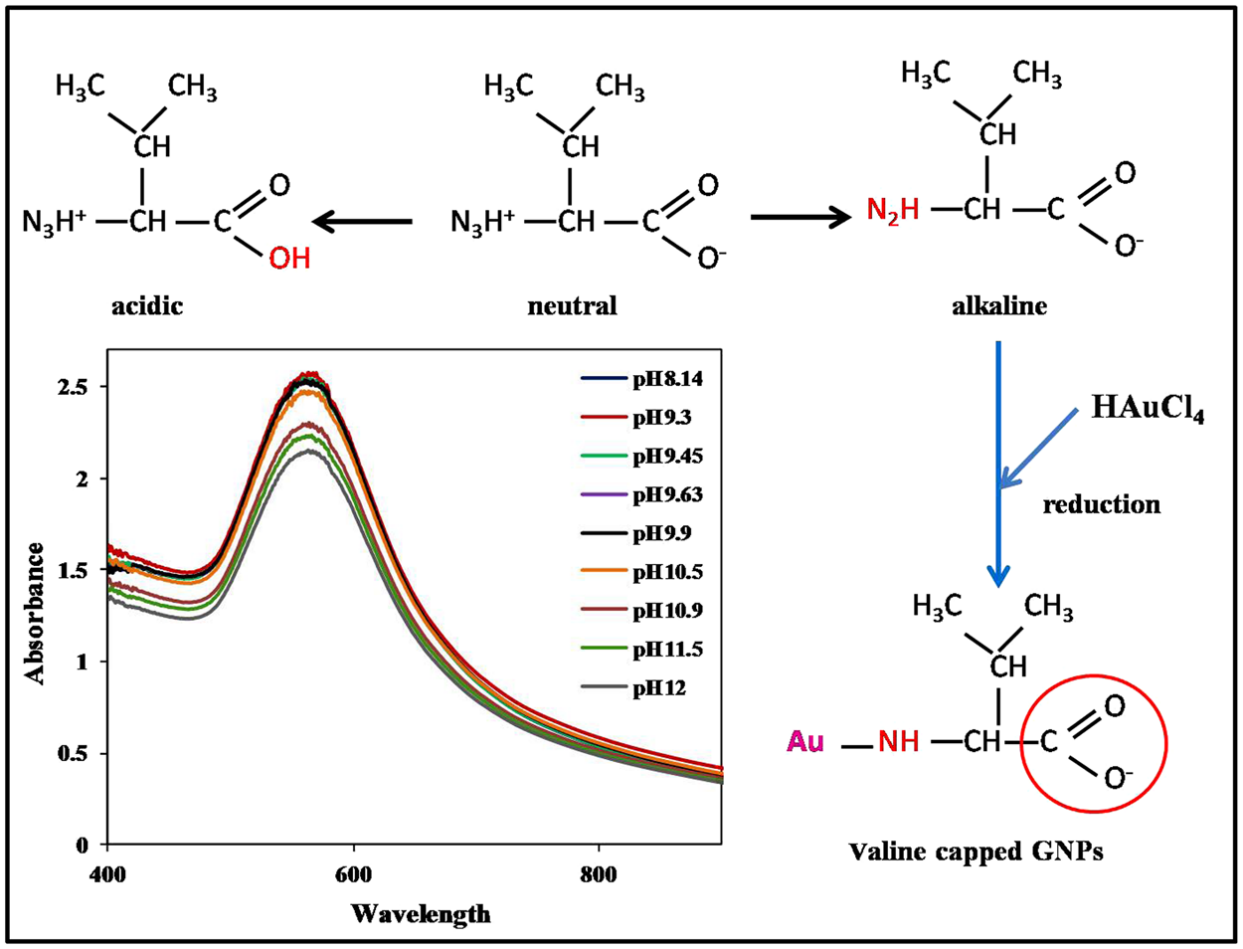
Schematic illustration of mechanism of GNP synthesis and stabilization by valine. Inset shows the effect of high alkalinity on valine capped GNPs.

Further, stability of valine-capped GNPs with increasing alkalinity of solution was determined by addition of increasing amount of NaOH solution. It was observed that the supplementation of high amount of NaOH to the already synthesized colloidal GNP solution, had no significant effect on the SPR peak of valine-capped GNPs (Inset in Fig. 3). This is because the addition of more –OH^−^ ions to synthesized GNPs above of pI of valine, (i.e. 6) have no effect on the surface charge of the biomolecule and they still remain negatively charged. This is in accordance with the results of Kumar *et al*., that suggests that valine capped GNPs possess a net negative charge on their surface beyond pH 6^23^. This confirms that GNP bind to valine molecule via –NH group and are stabilized by the –COO^−^ groups. The synthesized GNPs carry a net negative charge on their surface. Zeta potential analysis provided further information about high stability of valine capped GNP. The valine-capped GNPs reported in this work were highly stable and even variation of pH after GNP synthesis has no effect on their size, shape and aggregation state (Inset in Fig. 3). A schematic representation of the mechanism of reduction and stabilization of GNPs by valine is exemplified in Fig. 3.

### Selective colorimetric detection of metal ions

The use of GNPs as optical sensors is generally based on determination of changes in the SPR peak positions and colour of the assay mixture. The change occurs due of the target-analyte induced agglomeration/aggregation of nanoparticles. This effect relies on the selectivity of the functionalizing ligand/capping molecule attached to the GNP surface. In the current study, valine molecules attach to GNP surface through the –NH group. The free –COO^−^ groups present a net negative charge on the surface of nanoparticles, stabilizing the GNP particles. Addition of metal ion (present in polluted water sample) may induce the cross-linking of GNPs by the target metal ion by coordinate bonding leading to destabilization of GNP and hence agglomeration. If the reaction is selective to only one metal ion this may form basis of metal detection.

**Figure 4:**
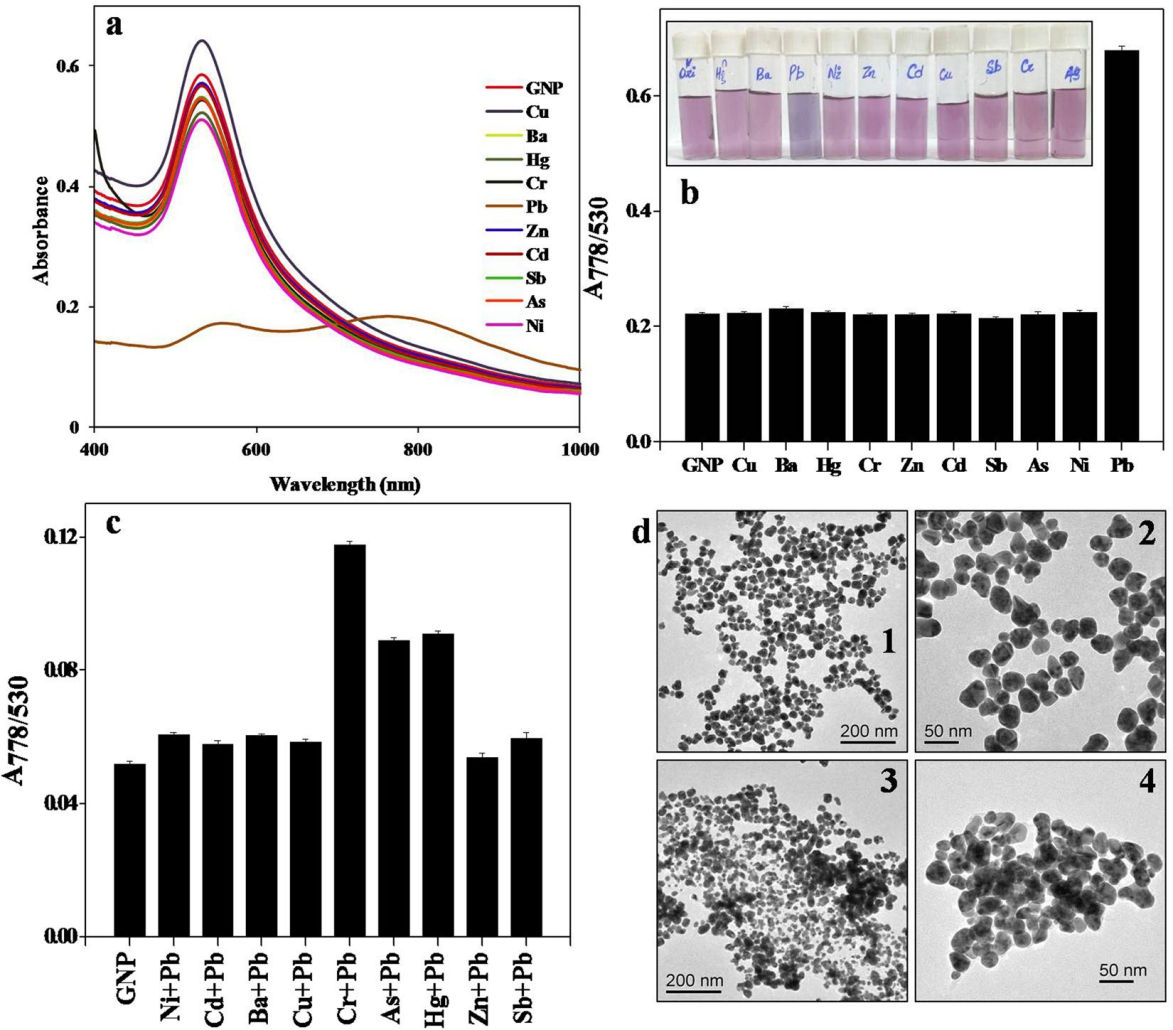
(**a**) Absorption spectra depicting the selectivity of valine- GNPs for Pb^2+^ ions. (**b**) Quantitaive analysis of selectivity of valine- GNPs. (**c**) Graph showing the absorption ratio obtained by treating valine- GNP with a comixture of 100 ppm of Pb^2+^ ion and 100 ppm of respective metal ion. (**d**) TEM images of valine- GNPs (1, 2) before treatment with Pb^2+^ ions (3, 4) after treatment with Pb^2+^ ions.

With this in mind, we treated the valine capped GNPs with 100 ppm of different metal ions (Cu^2+^, Ba^2+^, Hg^2+^, Cr^3+^, Pb^2+^, Zn^2+^, Cd^2+^, Sb^3+^, As^3+^, Ni^2+^) individually and recorded the absorption spectra of the assay solution. We observed that when GNPs were treated with Pb^2+^ ion solution, an immediate change in colour of the assay solution from the original violet colour to blue occured, while all other metal assay solutions retained the original colour of colloidal GNP (Fig. 4a). The absorption spectra also showed evident changes in the SPR peak of Pb^2+^ assay solution. The plasmon peak of colloidal GNP was originally situated at 530 nm. Except for Pb^2+^, the SPR peak of all other metal treated assay solution showed a negligible change and remained at the same position as that of untreated colloidal GNP. However, in case of Pb^2+^ assay solution, a new absorption peak appeared at higher wavelength region (778 nm) along with decrease in original SPR peak at 530 nm. The typical change in colour and absorption spectra of treated GNP solution are shown in Fig. 4b. Quantitative analysis of detection was performed by comparing the absorption ratio at 778 and 530 nm (*A*_778_/*A*_530_). This ratio represents the degree of aggregation, a representative of the relative quantity of aggregated and dispersed nanoparticles in the solution^24^. Comparative quantitative analysis of the tested metal assay solution suggested an apparently high absorption ratio for Pb^2+^ suggesting the maximum aggregation state, while all other metals had an absorption ratio similar to that of untreated GNP (Fig. 4b).

We also analyzed possible interference of other metal ions that may be present in the polluted water sample during Pb^2+^ ion detection using valine capped GNP. Of all the metal ions (Cu^2+^, Ba^2+^, Hg^2+^, Cr^3+^, Zn^2+^, Cd^2+^, Sb^3+^, As^3+^, Ni^2+^) tested in the study, only Cr^3+^, As^3+^ and Hg^2+^ affected the sensitivity of Pb^2+^ ion detection by valine capped GNPs (Fig. 4c **)**. Higher absorption ratio (A_778_/A_530_) was observed in case of the three metals (Cr^3+^, As^3+^, Hg^2+^) compared to original valine capped GNP. This indicates that their co-presence with Pb^2+^ ions triggers the aggregation of GNPs and induces a change in colour from violet to blue. But from Fig. 4b it is clear that in absence of Pb^2+^, the interfering ions like Cr^3+^, As^3+^ and Hg^2+^ do not react with GNP. Thus aggregation occurs only if Pb^2+^ is present in assay solution. Other tested ions did not interfere in the detection of Pb^2+^ and retained the original colour and SPR peak of colloidal GNPs. Thus, the valine-capped GNPs are capable of detecting Pb^2+^ ions selectively in presence of other metal ions also.

### Sensitivity of gold nanoparticles towards lead ions

**Figure 5:**
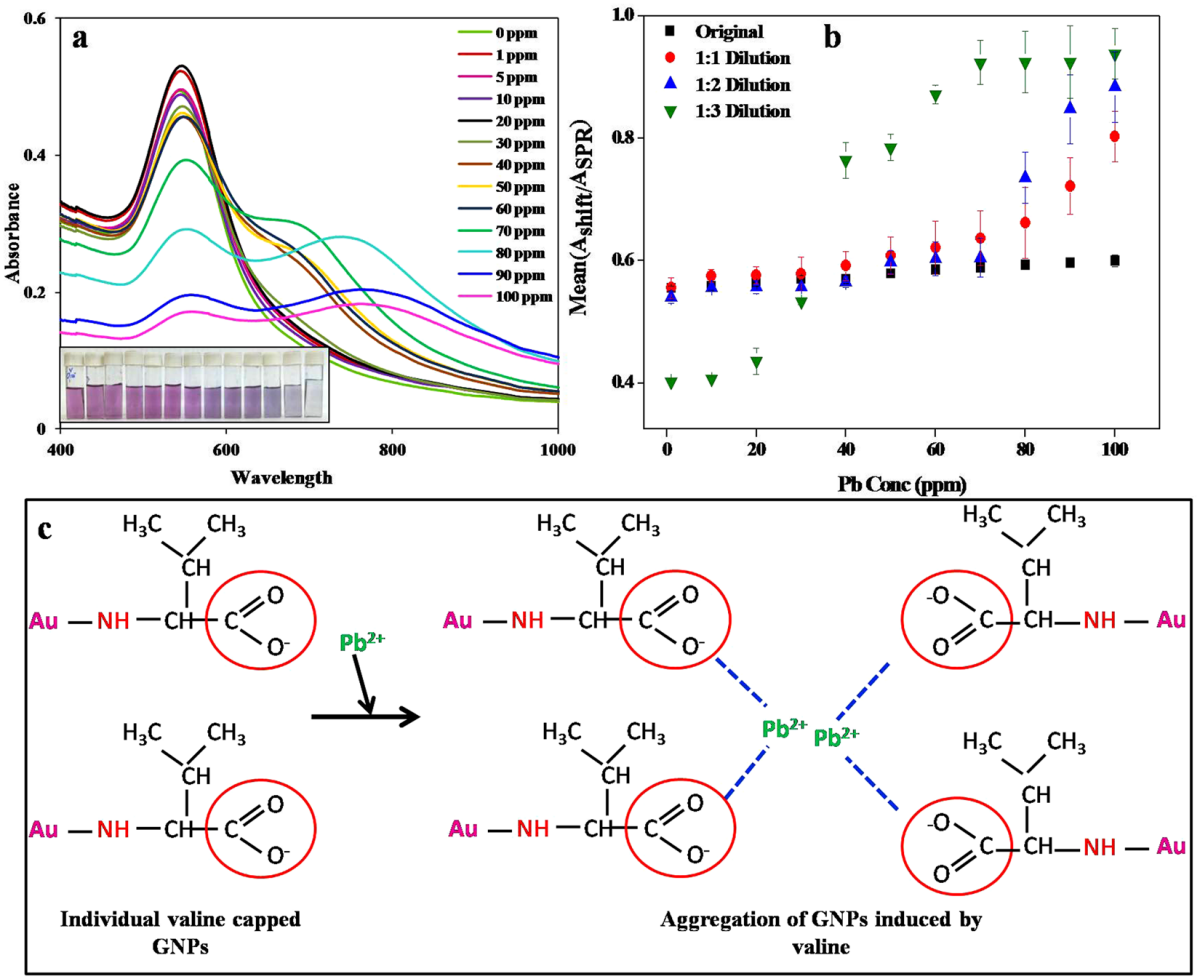
(**a**) Absorption spectra depicting the effect of increasing Pb concentration on SPR peak of valine- GNPs. (**b**) Quantitative analysis showing the effect of valine- GNP dilution on sensitivity of Pb detection (**c**) Schematic illustration of Pb detection using valine capped GNPs.

For practical applicability, it’s essential to determine the sensitivity and minimum detection limit (MDL) of the sensing system. Therefore, to quantitatively establish the dynamic detection concentration of Pb^2+^ ions by valine capped GNPs, experiments were performed using varying concentration of Pb^2+^ ions. With increasing concentration of Pb^2+^ ions, the intensity of original absorbance of GNP at 530 nm decreased, along with the appearance of new absorbance peak at higher wavelength range (780 nm). Furthermore, red shifting of the new longitudinal SPR peak was observed with increasing Pb^2+^ ion concentration. Distinct change in colour of colloidal GNP solution from purple, violet to blue was seen with progressively increasing concentration of Pb^2+^ ions. The variation in visual change in colour was consistent with the absorption spectroscopy (Fig. 5a). Interaction with Pb^2+^ ions leads to the formation of GNP agglomerates that results in a decrease in concentration of free mono-dispersed colloidal GNP. This results in redistribution of spectral intensity to longer wavelengths with decrease in absorption at 530 nm. Quantitative representation of agglomeration obtained by plotting Pb^2+^ concentration versus A_778_/A_530_ ratio showed a straight line graph with a very good curve fitting (R^2^ = 0.98) in the range of 1 to 100 ppm Pb^2+^ concentration.

The synthesized GNPs were diluted and then treated with Pb^2+^ ions to analyze the effect of dilution on detection sensitivity. It was observed that the diluted GNP solution showed a lower detection limit compared to that of original GNPs (Fig. 5b). Dilution results in well dispersed GNP particles with lower concentration. Hence, addition of even small amount of Pb^2+^ ions is enough to aggregate all the available GNPs. Gunupuru *et al*.^13^ reported detection of Pb^2+^ ions with a MDL of 10 ppm using calixarene functionalized GNPs, while in the present study the as-synthesized GNPs were capable of efficiently detecting Pb^2+^ ions with a MDL of 10 ppm using 1:3 diluted solution of GNPs.

TEM analysis of valine capped GNPs before and after treatment with Pb^2+^ ions clearly suggested the aggregation of synthesized particles. TEM micrographs of synthesized GNPs show individual separated particles (Fig. 4d (1 and 2)). The dispersed particles signify an efficient electrostatic barrier between the particles offered by the negative –COO^−^ groups that present an electrostatic repulsion against the vanderwaals attraction between the GNPs^25^, thus preventing agglomeration. On the other hand, TEM images of GNPs treated with 100 ppm of Pb^2+^ ions, after 5 minutes of incubation showed significant aggregation of particles (Fig. 4d (3 and 4). It is attributed that addition of Pb^2+^ to GNPs results in a change in colour of solution as well as distinct changes in the absorption spectra of the assay solution. Addition of Pb^2+^ ions to GNP results in chelating interaction of valine carboxylate group. Saunders *et al*. (2011) while studying the X-ray crystallography of Pb-Val molecule reported that Pb interacts with two valine carboxylate units and a water molecule with high affinity resulting in the chelation of Pb^2+^. As already stated the synthesized GNPs are electrostatically stabilized by the –COO^−^ functional groups of valine, hence interaction of Pb^2+^ ions with –COO^−^ groups leads to the destabilization of net negative charge on GNP surface consequently leading to agglomeration of particles. This forms the basis of high selective and sensitive colorimetric detection of Pb^2+^ ions by valine capped GNPs. The mechanism of interaction of Pb^2+^ ions with valine capped GNPs is illustrated in Fig. 5c.

Most of the earlier studies on colorimetric sensing of Pb^+2^ ions, reported the use of Pb-specific ligands for the detection process. In the present study, as-synthesized valine-GNPs showed the potential of Pb^2+^ ion detection. The method involves a rapid, one-step method for selective optical sensing of Pb^2+^ ion, even in presence of other major toxic heavy metal ions present in waste water. However development of an assay kit for such detection in environmental sample requires optimization of many parameters and several challenges need to be resolved. One of the major challenges is interference with sample matrix. Apart from the metal ions tested in the present study many other common ions including Cl, K, Na, Mg, P, Ca, Fe, sulphate, nitrate, phosphate, carbonate, bicarbonate ions are also found in waste water. Some organic molecules including pesticides, antibiotics, bacteria etc may also interfere in the detection process. Therefore, further studies on optimization of the optical detection of lead with respect to sensitivity in presence of these interfering matrix ions needs to be completed. These studies will assist in the development of heavy metal assay methods and kits for feasible on-site detection of lead ions in water/waste water samples with optimum efficiency and sensitivity.

## Conclusion

A cost effective and label free, one-pot approach for colorimetric detection of Pb^2+^ ions using valine-capped GNPs has been addressed in the present work. Detection of Pb^2+^ was based on an ion-dependent chelation mechanism. Treatment of valine-GNPs with Pb^2+^ ions resulted in rapid change in colour and absorption spectra due to the agglomeration of GNPs. The Pb^2+^ ions bind to the free –COO^−^ groups destabilizing the net charge on GNP surface. The synthesized GNPs showed selectivity for Pb^2+^ compared to other tested divalent cations. An enhancement in sensitivity of detection was observed when diluted solution of valine-GNPs was used. As the valine capped GNPs showed significant potential for detecting Pb^2+^ ions, the present sensing system holds tremendous potential for applications as cost effective and rapid colorimetric detection. This eliminates the functionalization step which is a major step reported in most of the other colorimetric studies.
